# PPARδ dysregulation of CCL20/CCR6 axis promotes gastric adenocarcinoma carcinogenesis by remodeling gastric tumor microenvironment

**DOI:** 10.1007/s10120-023-01418-w

**Published:** 2023-08-12

**Authors:** Yi Liu, Daoyan Wei, Yasunori Deguchi, Weiguo Xu, Rui Tian, Fuyao Liu, Min Xu, Fei Mao, Donghui Li, Weidong Chen, Lovie Ann Valentin, Eriko Deguchi, James C. Yao, Imad Shureiqi, Xiangsheng Zuo

**Affiliations:** 1 Department of Gastrointestinal Medical Oncology, The University of Texas MD Anderson Cancer Center, Houston, TX 77030 USA; 2 Department of Gastroenterology, Hepatology, and Nutrition, The University of Texas MD Anderson Cancer Center, Houston, TX 77030 USA; 3 Rogel Cancer Center and Department of Internal Medicine, Division of Hematology and Oncology, University of Michigan, Ann Arbor, MI 48109 USA

**Keywords:** Stomach neoplasms, PPARdelta, CCL20/CCR6, Immunosuppression, Biomarkers

## Abstract

**Background:**

Peroxisome proliferator-activated receptor delta (PPARδ) promotes inflammation and carcinogenesis in many organs, but the underlying mechanisms remains elusive. In stomachs, PPARδ significantly increases chemokine Ccl20 expression in gastric epithelial cells while inducing gastric adenocarcinoma (GAC). CCR6 is the sole receptor of CCL20. Here, we examine the role of PPARδ–mediated Ccl20/Ccr6 signaling in GAC carcinogenesis and investigate the underlying mechanisms.

**Methods:**

The effects of PPARδ inhibition by its specific antagonist GSK3787 on GAC were examined in the mice with villin-promoter–driven PPARδ overexpression (*Ppard*^*TG*^). RNAscope Duplex Assays were used to measure Ccl20 and Ccr6 levels in stomachs and spleens. Subsets of stomach-infiltrating immune cells were measured via flow cytometry or immunostaining in *Ppard*^*TG*^ mice fed GSK3787 or control diet. A panel of 13 optimized proinflammatory chemokines in mouse sera were quantified by an enzyme-linked immunosorbent assay.

**Results:**

GSK3787 significantly suppressed GAC carcinogenesis in *Ppard*^*TG*^ mice. PPARδ increased Ccl20 level to chemoattract Ccr6^+^ immunosuppressive cells, including tumor-associated macrophages, myeloid-derived suppressor cells and T regulatory cells, but decreased CD8^+^ T cells in gastric tissues. GSK3787 suppressed PPARδ–induced gastric immunosuppression by inhibiting Ccl20/Ccr6 axis. Furthermore, Ccl20 protein levels increased in sera of *Ppard*^*TG*^ mice starting at the age preceding gastric tumor development and further increased with GAC progression as the mice aged. GSK3787 decreased the PPARδ-upregulated Ccl20 levels in sera of the mice.

**Conclusions:**

PPARδ dysregulation of Ccl20/Ccr6 axis promotes GAC carcinogenesis by remodeling gastric tumor microenvironment. CCL20 might be a potential biomarker for the early detection and progression of GAC.

**Supplementary Information:**

The online version contains supplementary material available at 10.1007/s10120-023-01418-w.

## Introduction

Gastric cancer (GC) is the fifth most common malignancy and the fourth most lethal cancer worldwide, with a 5-year survival rate of 5%-10% in advanced stages [1, 2]. Novel GC therapies and reliable biomarkers for the early detection and progression of GC are urgently needed to improve the outcomes of GC patients.

Intensive research work has been performed to identify therapeutic targets for GC interventions, but progress has been limited, partially because of a lack of suitable in vivo preclinical models. Peroxisome proliferator-activated receptor delta (PPARδ, encoded by *PPARD*) is a nuclear transcriptional factor that regulates physiologic and pathophysiologic processes, especially those involved in cell stemness, inflammation, and tumorigenesis [3–10]. PPARδ is upregulated in human GC tissues, and its expression is associated with human GC grades and stages, suggesting PPARδ is a potential therapeutic target for GC. Moreover, we recently reported that transgenic PPARδ overexpression in villin-expressing gastric progenitor cells (VGPCs) [11] in mice (termed *Ppard*^*TG*^ mice) activates and transforms quiescent VGPCs and induces progression of gastric tumorigenesis from metaplasia to dysplasia, and finally to invasive intestinal-type gastric adenocarcinoma (GAC) that is associated with severe gastric chronic inflammation, faithfully recapitulating the pathogenic features of human intestinal-type GAC, the most common type of GC [8]. Chronic inflammation modulates tumor microenvironment (TME) to promote gastric tumor immune evasion and subsequently gastric carcinogenesis [12]. PPARδ expression in VGPCs triggered chronic gastric inflammation [8]; whether and how PPARδ modulates gastric TME to promote gastric carcinogenesis remains unknown.

The immunosuppressive TME (iTME) plays a pivotal role in promoting carcinogenesis [13, 14]. The populations of myeloid-derived suppressor cells (MDSCs) and tumor-associated macrophages (TAMs) expand during tumorigenesis to promote iTME development and tumorigenesis including GC [15–18]. Our previous RNA-seq findings showed that the top three differentially altered pathways regulated by PPARδ were all related to immune modulatory mechanisms, and CCL20 was the chemokine most upregulated by transgenic PPARδ expression in VGPCs of *Ppard*^*TG*^ mice [8]. The sole receptor of CCL20 is CCR6 [19]. Accumulating data show that the alteration of CCL20/CCR6 axis promotes cancer progression in various types of tumors including GC [20, 21]. However, whether and how PPARδ upregulation of CCL20 in gastric epithelial cells orchestrates an iTME and subsequentially promotes GAC tumorigenesis in *Ppard*^*TG*^ mice remains unknown. Hence, in this study, we aimed to examine whether transgenic PPARδ expression in VGPCs orchestrates the gastric iTME to promote GAC tumorigenesis via upregulating the Ccl20/Ccr6 axis; whether specially targeted inhibition of PPARδ suppresses GAC tumorigenesis by inhibiting Ccl20/Ccr6 axis to significantly reverse the iTME; and whether circulating CCL20 in sera can serve as a potential biomarker for the early detection and tumor progression of GAC.

## Materials and methods

### Animals

*Ppard*^*TG*^ mice were produced from two independent founders (*Ppard*^*TG*^-1 and *Ppard*^*TG*^-2) that were generated at The University of Texas MD Anderson Cancer Center Genetically Engineered Mouse Facility by pronuclear injection of mouse *Ppard* expression construct under the control of a villin promoter (p12.4Kvill–*Ppard*) into fertilized FVB oocytes [22]. The two *Ppard*^*TG*^ mouse lines were found to exhibit similar PPARδ expression levels and display similar phenotypes [8, 22]. Therefore, all subsequent experiments were performed using randomly selected *Ppard*^*TG*^-1 and *Ppard*^*TG*^-2 mice, designated as *Ppard*^*TG*^ hereafter. The mice were housed with a dark/light cycle of 12 h, ambient temperature of 22 °C, and humidity of 30%-70%.

### Mouse treatment with PPARδ antagonist GSK3787 diet and evaluation of gastric tumorigenesis

*Ppard*^*TG*^ mice and their sex- and gender- matched WT littermates at age 6–8 weeks were fed either control diet (#TD.110161: 2019 Teklad Global 19% Protein Rodent Diet, Envigo) or the same control diet but with adding GSK3787 (#G7423, Sigma-Aldrich; customized diet, Envigo, 200 mg/kg) for 44 weeks (n = 6–10 mice per group). Then, the mice were euthanized, and the stomach of each mouse was removed, weighed, photographed, and grossly inspected for tumor formation. Half of the stomach from each mouse was harvested for RNA and protein analyses, and the other half was put in 10% neutral formalin for further sectioning analysis.

### Mouse stomach tissue histology

Formalin-fixed gastric tissue samples were embedded in paraffin and sectioned onto slides at 5 μm thick and then stained with hematoxylin and eosin. Digital hematoxylin and eosin staining slides for mouse gastric tissues were scanned with Aperio AT2 (Leica Biosystems), and the scanned images were captured with Aperio ImageScope software (v12.3.3.5048). Histologic assessment for gastric tumor lesions was performed with the support of an experienced rodent pathologist in the MD Anderson veterinary pathology services facility.

### In situ hybridization staining of RNAscope duplex assay

In situ hybridization staining of RNAscope Duplex Assay was performed according to the manufacturer’s manual (RNAscope 2.5 HD Duplex assay, #322500-QKG, Bio-Techne ACDBio). Briefly, the freshly cut 5 µm-sections were deparaffinized and treated with 3% H_2_O_2_. The slides were then boiled in RNAscope specific target retrieval reagent (Bio-Techne ACDBio), washed with 100% ethanol, dried, and incubated with protease reagent in the humidity control tray in the HybEZ oven (Bio-Techne ACDBio). Next, the hybridization was performed in the HybEZ oven using the following probes: mouse *Ccl20* (#434051, Bio-Techne ACDBio) with mouse *villin* (#463301-C2, Bio-Techne ACDBio) or with mouse *Ccr6* (#424461-C2, Bio-Techne ACDBio). After the hybridization, the slides were washed, and the signals were amplified by using RNAscope 2.5 HD Duplex Detection Kit (#322500, Bio-Techne ACDBio), followed by Mayer’s hematoxylin staining. RNA in situ hybridization intensity was scored as follows: the red dots as positive staining per cell for each slide were counted under microscope with 10X magnification and scored and averaged for five random fields as follows: 0 (no staining or < 1 dot/10 cells), 1 + (1–3 dots/cell), 2 + (4–10 dots/cell, very few dot clusters), 3 + (> 10 dots/cell, and < 10% dot clusters), or 4 + (> 10 dots/cell, and ≥ 10% dot clusters) according to the manufacturer’s guideline (SOP 45–006, Bio-Techne ACDBio).

### Statistical analysis

Statistical significance was determined by the unpaired Student *t*-test or analysis of variance (one-way ANOVA with Tukey’s multiple comparisons test or two-way ANOVA with Šídák’s multiple comparisons test). Kaplan–Meier survival analysis and the log-rank test were used to compare survival outcomes. The statistical significance of the correlation of two factors was determined by Spearman correlation analysis. All tests were two-sided, and significance was defined at *P* < 0.05. Data were analyzed using SAS software, version 9.4 (SAS Institute) or GraphPad Prism 9 (GraphPad Software). Values presented are mean ± standard error of the mean (SEM) unless otherwise specified (*P < 0.05, **P < 0.01, ***P < 0.001, and ****P < 0.0001).

## Results

### PPARδ expression is negatively associated with overall survival (OS) and progression-free survival (PFS) in a large cohort of GC patients

**Fig. 1:**
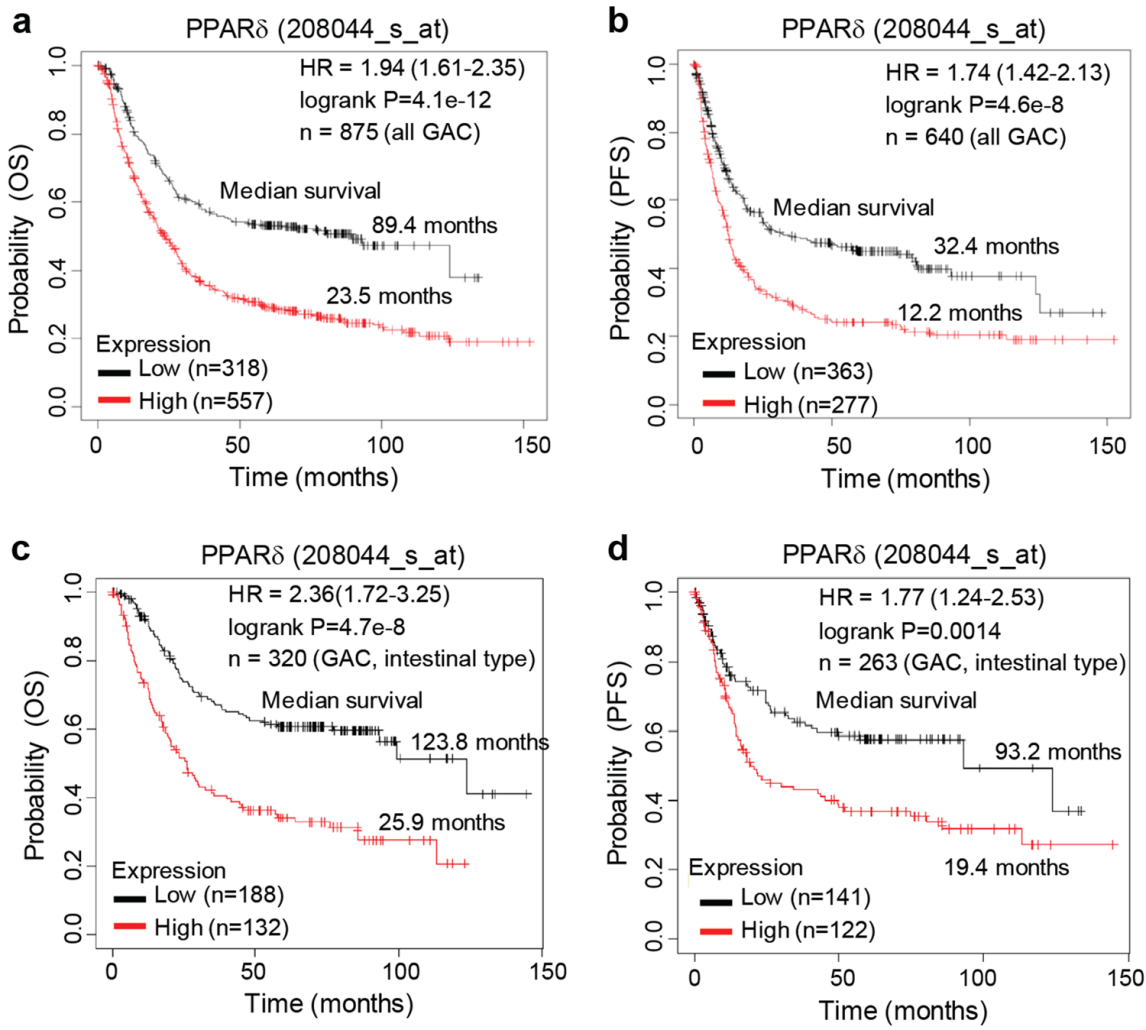
Kaplan–Meier plots of OS and PFS for GAC (all types) and GAC (intestinal-type) patients with low vs. high PPARδ expression. **a, b** OS and PFS for patients with GAC of all types. **c, d** OS and PFS for patients with GAC (intestinal type). *P* values were calculated by log-rank test. HR (95% confidence interval) was from Cox regression using the high expression group as reference. *HR* hazard ratio

PPARδ is upregulated in GC tissues and its expression is associated with GC grades and stages in patients with GC [8]. To further evaluate the impact of PPARδ on the patients’ survival, we searched a large cohort of GC patients in the Kaplan–Meier Plotter public database [23] and found that PPARδ mRNA expression levels measured with a specific PPARδ probe (208044_s_at) were negatively associated with the GC patients’ OS and PFS: among the 875 evaluated GC patients, those with low PPARδ mRNA expression had longer median OS (89.4 months [n = 318]) and PFS (32.4 months [n = 363]) than those with high PPARδ mRNA expression (OS: 23.5 months [n = 557] and PFS: 12.2 months [n = 277]) (Fig. 1a, b). Similarly, among the 320 patients with GAC (intestinal type), those with low PPARδ mRNA expression had longer median OS (123.8 months [n = 188]) and PFS (93.2 months [n = 141]) than those with high PPARδ mRNA expression (OS: 25.9 months [n = 132] and PFS: 19.4 months [n = 122]) (Fig. 1c, d).

### PPARδ-specific antagonist GSK3787 suppresses GAC progression in mice

**Fig. 2:**
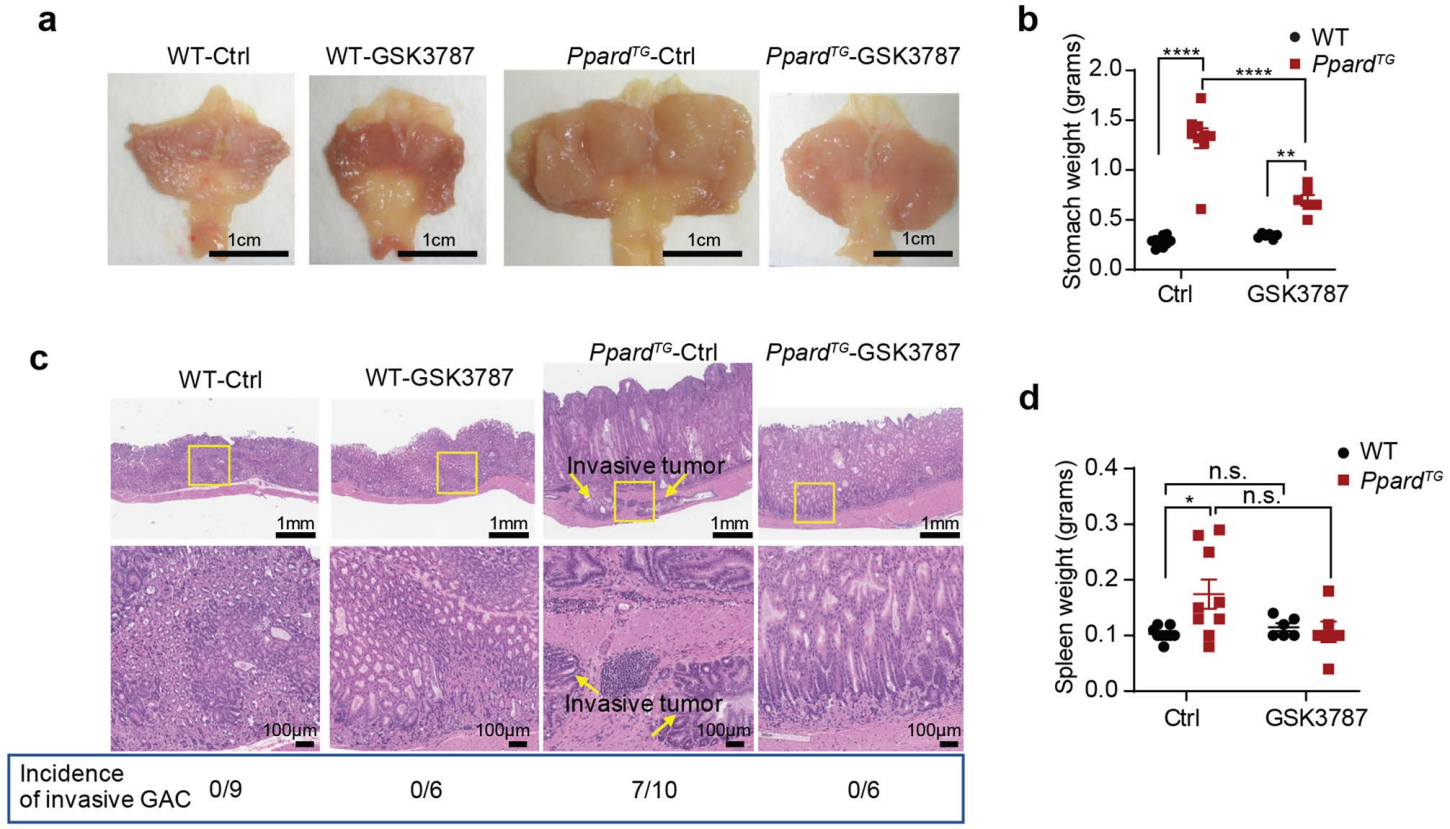
PPARδ–specific antagonist GSK3787 suppressed GAC progression in *Ppard*^*TG*^ mice. *Ppard*^*TG*^ mice and WT littermates at age 6 weeks (6–10 mice per group) were fed a GSK3787 (200 mg/kg) or control (Ctrl) diet for 44 weeks and then euthanized. **a** Gross examination of the stomachs. **b** Weights of stomachs. **c** Photomicrographs of hematoxylin and eosin–stained sections of the stomachs showing locally invasive adenocarcinoma into the muscle layers of stomach walls and marked transmural infiltrations of many lymphocytes in *Ppard*^*TG*^ mice fed a control diet in comparison with non-invasive tumors in *Ppard*^*TG*^ mice fed a GSK3787 diet. None of the WT mice developed gastric tumors. Yellow arrows indicate invasive tumors. **d** Weights of spleens. Data for** b** and **d** are mean ± SEM. * *P* < 0.05; ** *P* < 0.01; **** *P* < 0.0001; n.s.: not significant; by two-way ANOVA followed by Šídák's multiple comparisons test

Our previous finding that PPARδ overexpression in VGPCs in mice induces large and invasive GAC accompanied with severe gastric chronic inflammation [8] prompted us to explore whether specifically targeting PPARδ is effective for GAC interventions, given that PPARδ antagonists have been developed. GSK3787 is a synthetic and irreversible antagonist of PPARδ that covalently binds to a cysteine residue in the ligand-binding domain of PPARδ [24]. In this study, we fed *Ppard*^*TG*^ mice at age 6–8 weeks the GSK3787 (200 mg/kg) or a control diet for 44 weeks. We found that this GSK3787 treatment significantly inhibited GAC carcinogenesis by decreasing stomach weights and inhibiting inflammatory cells’ infiltration, and GAC progression (Fig. 2a–c). A total of 7 of 10 *Ppard*^*TG*^ mice fed the control diet developed locally invasive GAC, but none of the sex- and age-matched *Ppard*^*TG*^ mice fed the GSK3787 diet had invasive GAC (Fig. 2c). The weights of spleens from *Ppard*^*TG*^ mice were significantly higher than those in control WT mice, but GSK3787 treatment had no effects on spleen weights in either WT or *Ppard*^*TG*^ mice (Fig. 2d). To assess the side effects of this long-term treatment with GSK3787, we evaluated liver and kidney function in the mice used for Fig. 2 by measuring serum levels of total protein, albumin, globulin, ALP, ALT and AST to monitor liver function and BUN and creatinine to monitor kidney function. While, unsurprisingly, *Ppard*^*TG*^ mice had lower serum protein levels, higher ALP and higher ALT levels than WT mice (Table S1), GSK3787 had no significant effects on liver or kidney function for WT and *Ppard*^*TG*^ mice (WT–Ctrl vs WT–GSK3787 or *Ppard*^*TG*^–Ctrl vs *Ppard*^*TG*^–GSK3787), indicating that GSK3787 was well tolerated in this preclinical study. These results further demonstrate the critical role of PPARδ in GAC tumorigenesis and suggest potential translational implications of targeting altered PPARδ for GAC interventions.

### PPARδ’s upregulation of Ccl20 chemoattracts, while GSK3787 inhibits, Ccr6^+^ immune cells infiltrating into gastric tissues of the mice

**Fig. 3:**
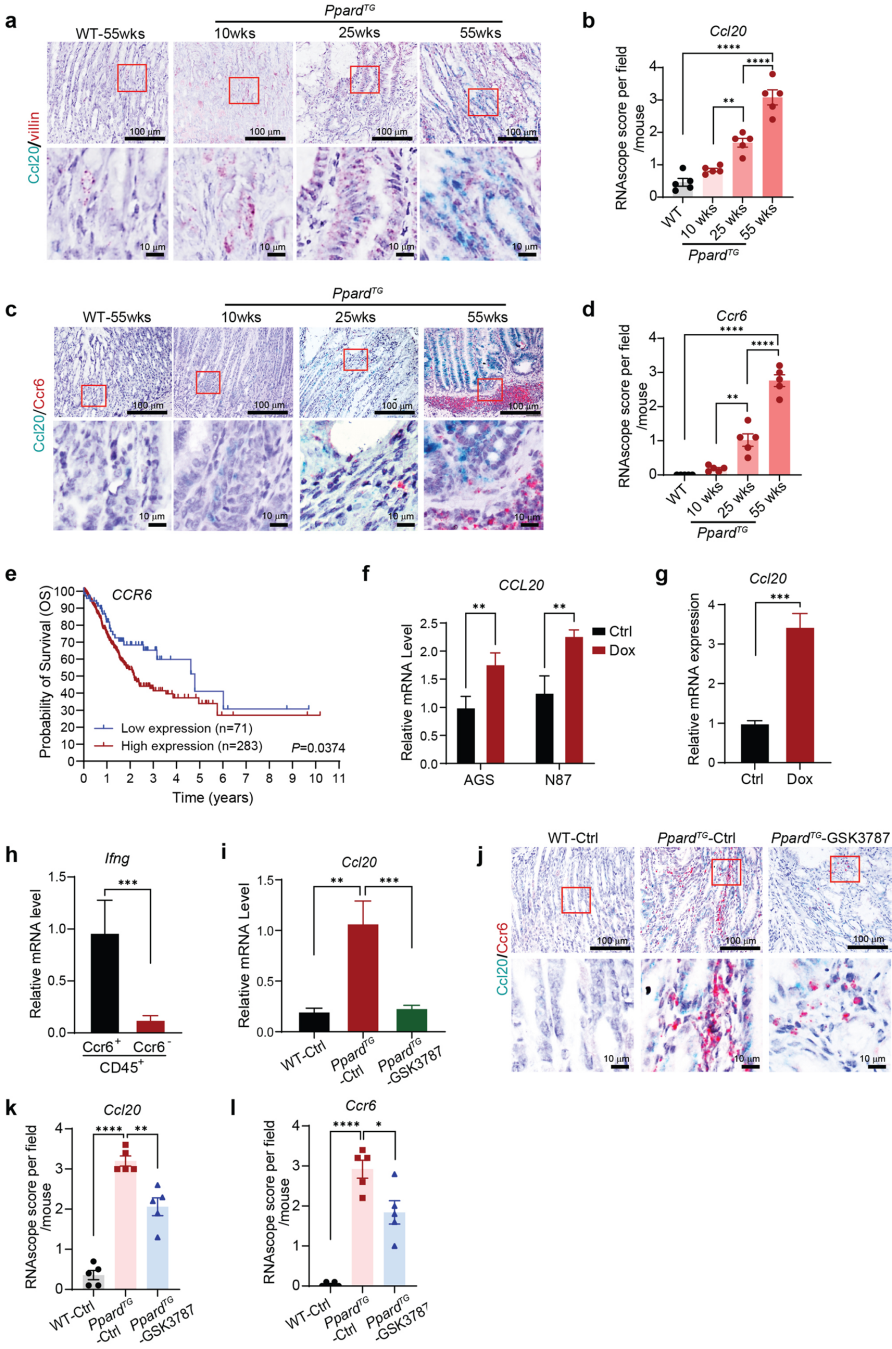
PPARδ upregulation of Ccl20 chemoattracted, but GSK3787 inhibited Ccr6^+^ immune cells infiltrating into gastric tissues. **a**–**d** Representative images of RNAscope Duplex Assay for *Ccl20* and *villin* (**a**) and the quantitative scores of *Ccl20* (**b**) or for *Ccl20* and *Ccr6* (**c**) and the quantitative scores of *Ccr6* (**d**) in the stomachs of *Ppard*^*TG*^ and WT mice at the indicated ages. **e** Overall survival of GC patients with low vs. high *CCR6* mRNA expression from analysis of Stomach Adenocarcinoma (STAD) RNA-seq data from TCGA cancer database. P value was calculated by Mantel-Cox test. **f**, **g** *CCL20* mRNA expression was measured by RT-qPCR in AGS and N87 human GC (**f**) and mouse GC (**g**) cells transduced with human or mouse Dox-inducible PPARδ expression lentivirus with Doxycycline (2 µg/mL) or its dissolvent treatment for 48 h. **h** *Ifng* mRNA expression levels measured by RT-qPCR in flow cytometry–sorted Ccr6^+^ and Ccr6^−^ stomach–infiltrating inflammatory immune cells from *Ppard*^*TG*^ mice at age 50 weeks. **i**–**l** The *Ppard*^*TG*^ mice and their WT littermates at 6–8 weeks were fed either a GSK3787 (200 mg/kg) or a control diet for 44 weeks and then euthanized as described in Fig. 2. **i** Gastric epithelial cells were scraped and examined for *Ccl20* mRNA expression measured by RT-qPCR. **j**–**l** Representative images of in situ hybridization staining of RNAscope Duplex Assay for *Ccl20* and *Ccr6* (**j**) and the quantitative scores of *Ccl20* (**k**) and *Ccr6* (**l**) in stomachs of the indicated mice. Data are mean ± SEM for **b**, **d**, **i**, **k** and **l**. * *P* < 0.05; ** *P* < 0.01; *** *P* < 0.001; ****P < 0.0001; by one-way ANOVA followed by Tukey’s multiple comparisons test. Data are mean ± SD for** f**–**h**. ** *P* < 0.01; *** *P* < 0.001; by unpaired *t* test

GAC development was strongly associated with chronic gastric inflammation in *Ppard*^*TG*^ mice [8], but the underlying mechanisms remained elusive. We previously reported that Ccl20 was the chemokine most markedly upregulated by transgenic PPARδ expression in VGPCs [8]. CCR6 is a non-promiscuous chemokine receptor that has only one known chemokine ligand, CCL20, and commonly exists on T cells and myeloid cells [19], the majority of gastric tumor–infiltrating immune cells in *Ppard*^*TG*^ mice [8]. To understand the biological significance and underlying mechanisms of PPARδ-mediated Ccl20 upregulation in the regulation of the gastric inflammation and GAC development, we performed in situ hybridization staining of RNAscope Duplex Assay for *Ccl20* with *villin*, or with *Ccr6* in stomachs and spleens of *Ppard*^*TG*^ mice and their WT littermates and found the following: (1) spleens of both *Ppard*^*TG*^ and WT mice had enriched *Ccr6*^+^ immune cells but no detectable *Ccl20* mRNA expression (Fig. S1a). (2) Transgenic PPARδ overexpression in VGPCs significantly increased expansion and transformation of double *Ccl20*^+^ and *villin*^+^ VGPC cells and eventually drove these cells to form GAC as the *Ppard*^*TG*^ mice aged from 10 to 55 weeks (Fig. 3a, b). (3) As *Ccl20* expression gradually increased in VGPCs, this transgenic PPARδ overexpression also gradually increased numbers of *Ccr6*^+^ immune cells infiltrating into the stomach, particularly into the gastric epithelial crypts or into gastric submucosa between the bottom of the gastric mucosa and the muscle layer in *Ppard*^*TG*^ mice from age 10 weeks to 55 weeks (Fig. 3c, d). CCR6 protein expression is significantly associated with metastasis, stage, and poor prognosis of GC [25]. We also retrieved and analyzed Stomach Adenocarcinoma (STAD) RNA-seq data from TCGA cancer database using The Human Protein Atlas’s Kaplan–Meier survival analysis tool and found that *CCR6* mRNA expression was negatively associated with GC patient overall survival (Fig. 3e). In an in vitro study, Doxycycline (Dox)–induced human PPARδ overexpression in human GC cell lines AGS and N87 (Fig. S1b) and Dox–induced mouse PPARδ overexpression in mouse GC cells generated from GAC of 55-week-old *Ppard*^*TG*^ mice in our laboratory [8] (Fig. S1c) significantly increased *CCL20* mRNA expression in these cells (Fig. 3f, g), indicating that PPARδ transcriptionally upregulated CCL20 in human and mouse GC cells. Ccr6^+^ and Ccr6^−^ gastric tumor–infiltrating CD45^+^ immune cells from *Ppard*^*TG*^ mice were then sorted by flow cytometry. Ccr6^+^CD45^+^ immune cells had higher *Ifng* mRNA expression than Ccr6^−^CD45^+^ cells did (Fig. 3h), indicating that Ccl20 might chemoattract Ccr6^+^CD45^+^ cells via the Ccl20/Ccr6 axis into the gastric tissues to secrete Ifng, a strong pro-inflammation cytokine, to promote chronic inflammation and GAC development, which further supports our previous findings [8].

In sharp contrast, GSK3787 significantly inhibited PPARδ–induced Ccl20 upregulation (Fig. 3i–k) and infiltration of Ccr6^+^ immune cells into stomachs in *Ppard*^*TG*^ mice (Fig. 3j, l) while suppressing GAC progression (Fig. 2). These data substantiate the critical role of PPARδ-Ccl20/Ccr6^+^ immune cell signaling alteration in chronic gastric inflammation and GAC development.

### PPARδ promotes but GSK3787 inhibits gastric immune suppression in Ppard^TG^ mice

**Fig. 4:**
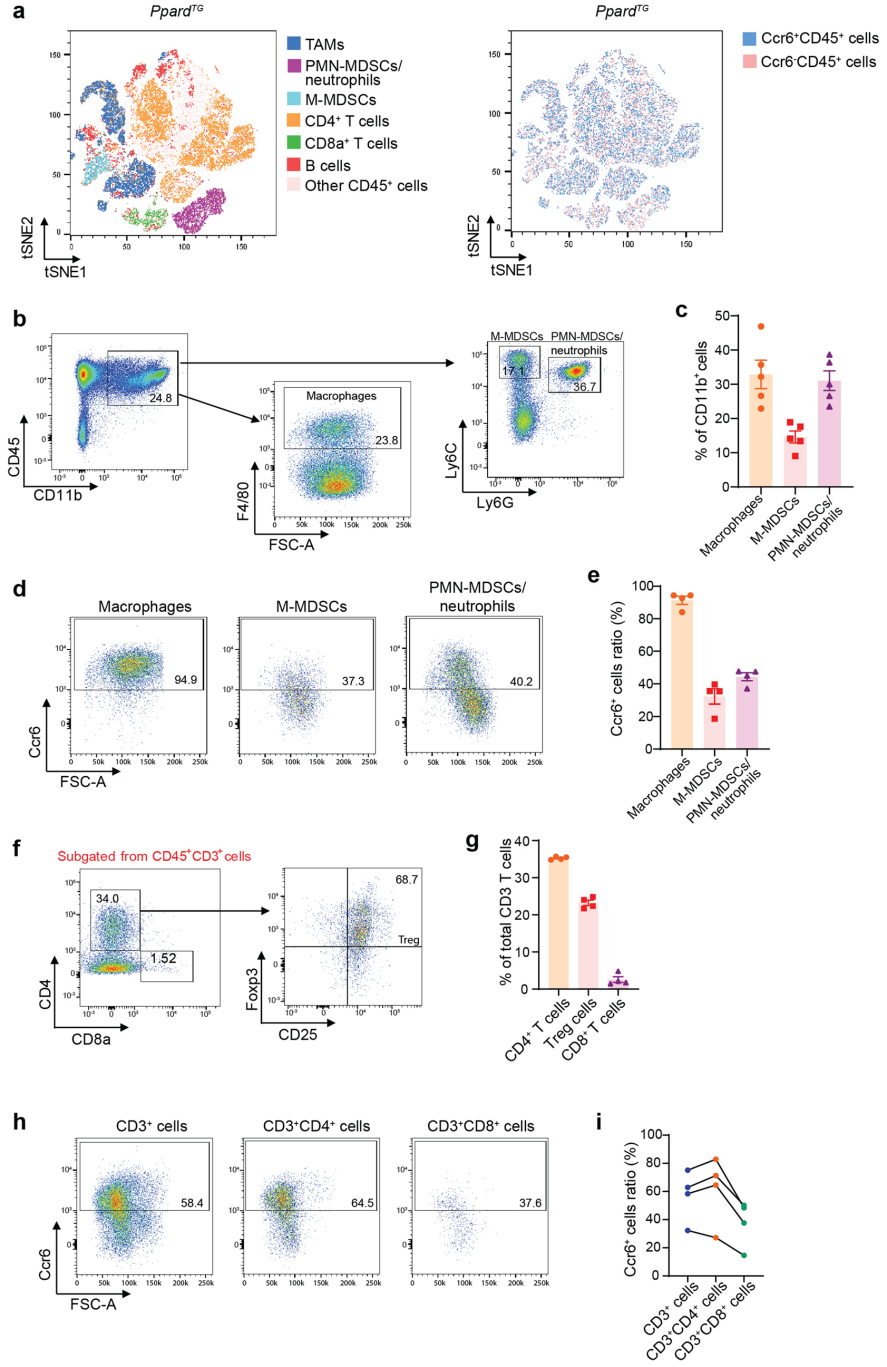
Subsets of total and Ccr6^+^ immune cells were profiled and quantified in stomachs of *Ppard*^*TG*^ mice. The *Ppard*^*TG*^ and WT mice at age 50 weeks were euthanized, and the stomachs were harvested and examined for immune cell profiling by flow cytometry. The WT mice had extremely low abundance of CD45^+^ immune cells in gastric tissues that precluded meaningful analyses. **a** Representative immune cell profiling by FlowJo-tSNE (t-distributed stochastic neighbor embedding) of stomach-infiltrating CD45^+^ cells based on parameters of CD45, CD11b, Ly6C, Ly6G, F4/80, CD3, CD4, CD8a and CCR6. The different colors indicated different immune cell subgroups, including macrophages, M-MDSCs, PMN-MDSCs/neutrophils, CD4^+^ T cells, CD8^+^ T cells, and B cells in *Ppard*^*TG*^ mice. **b**, **c** Representative flow cytometry images of stomach–infiltrating macrophages, M-MDSCs and PMN-MDSCs/neutrophils (**b**) and their percentages out of CD45^+^CD11b^+^ myeloid cells (**c**) in *Ppard*^*TG*^ mice (n = 5 mice). **d**, **e** Representative flow cytometry images (**d**) and ratios (**e**) of stomach–infiltrating Ccr6^+^ macrophages, Ccr6^+^ M-MDSCs, and Ccr6^+^ PMN-MDSCs/neutrophils in *Ppard*^*TG*^ mice (n = 4 mice). **f**, **g** Representative flow cytometry images (**f**) and percentages (**g**) of stomach–infiltrating CD4^+^ T, Tregs and CD8^+^ T out of CD3^+^ T cells in *Ppard*^*TG*^ mice. **h**, **i** Representative flow cytometry images (**h**) and ratios (**i**) of stomach– infiltrating Ccr6^+^ CD3^+^, Ccr6^+^CD4^+^ and Ccr6^+^CD8^+^ T cells in *Ppard*^*TG*^ mice. Data are mean ± SEM

We next performed immune cell profiling to further characterize the subpopulations of the stomach-infiltrating immune cells that promote chronic gastric inflammation and GAC development. Stomachs of WT mice had very limited immune cells that were insufficient for further immune cell profiling [8]; thus, we performed multiple panels of immune cell profiling by flow cytometry on the gastric corpus tissues of *Ppard*^*TG*^ mice at age 50 weeks, when GAC developed, to define subsets of immune cells in the stomachs. The majority of stomach-infiltrating CD45^+^ immune cells from *Ppard*^*TG*^ mice were CD11b^+^ myeloid cells and CD3^+^ T cells (Fig. S2a). The immune cell phenotype of *Ppard*^*TG*^ tumors analyzed by FlowJo-tSNE (t-distributed stochastic neighbor embedding) showed more abundant tumor-associated macrophages (TAMs), polymorphonuclear (PMN)-MDSCs/neutrophils, monocytic (M)-MDSCs, and CD4^+^ T cells than CD8^+^ T cells and B cells (Fig. 4a [left]), as well as more abundant CCR6^+^ immune cells than CCR6^−^ immune cells (Fig. 4a [right]). 32.86% ± 4.15% (mean ± SEM, hereafter unless otherwise specified), 14.60% ± 1.74%, and 31.04% ± 2.87% of stomach-infiltrating CD11b^+^ myeloid cells were TAMs (CD11b^+^F4/80^+^), M-MDSCs (CD11b^+^Ly6C^high^Ly6G^−^), and PMN-MDSCs/neutrophils (CD11b^+^Ly6C^low^Ly6G^+^), respectively (Fig. 4b, c). Not surprisingly, 68.80% ± 4.30% of CD45^+^ cells, 75.34% ± 5.99% of CD45^+^CD11b^+^ cells (Fig. S2b, c), 91.38% ± 2.49% of TAMs, 32.30% ± 4.64% of M-MDSCs and 44.33% ± 2.49% of PMN-MDSCs/neutrophils (Fig. 4d, e) in the stomachs of *Ppard*^*TG*^ mice were Ccr6^+^. Further analyses of CD3^+^ T subsets showed that the stomach-infiltrating CD3^+^ T cells had 1) 35.25% ± 0.21% CD4^+^ and 23.23% ± 0.72% T regulatory cells (Tregs, CD4^+^CD25^+^Foxp3^+^), indicating that Tregs were the majority of stomach infiltrating CD4^+^ T cells; 2) only 2.54% ± 0.77% CD8^+^ T cells, and 3) low ratios of CD8^+^ T cells to CD4^+^ T cells (mean ± SD: 0.07 [1:14 for CD8^+^: CD4^+^] ± 0.04) in *Ppard*^*TG*^ mice (Fig. 4f, g). The ratios of Ccr6^+^ cells of CD3^+^ (57.18% ± 9.01%) and CD4^+^ (61.48% ± 12.04%) were higher than those of CD8^+^ T cells (37.68% ± 8.17%) in the stomach of every examined mouse (Fig. 4h, i).

**Fig. 5:**
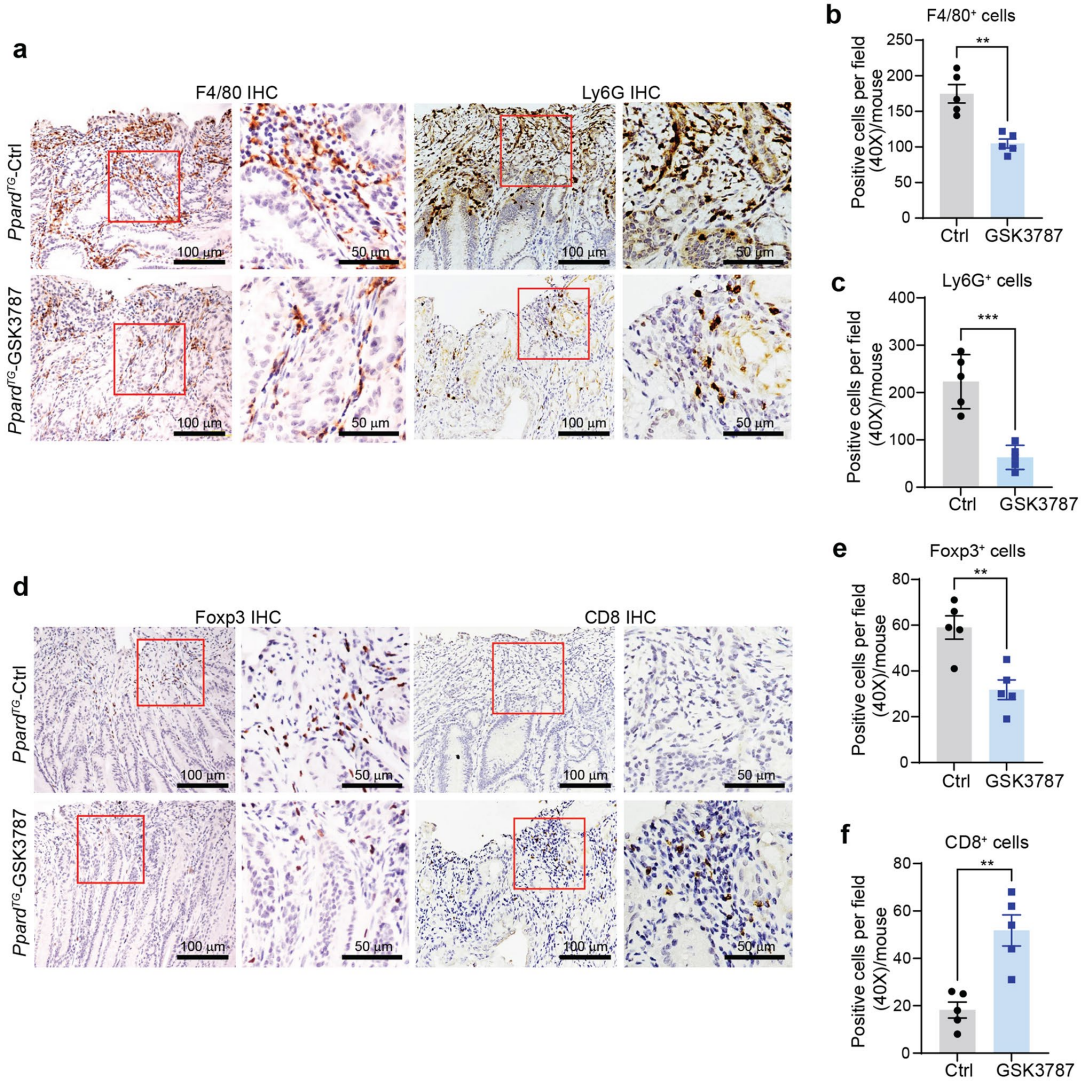
GSK3787 inhibited PPARδ-mediated immune suppression in *Ppard*^*TG*^ mice. **a**–**c** Representative immunohistochemistry images (**a**) and quantitative data (**b**, **c**) for macrophages (F4/80^+^) and PMN-MDSCs/neutrophils (Ly6G^+^) in the stomachs of *Ppard*^*TG*^ mice fed a GSK3787 or control (Ctrl) diet as described in Fig. 2 (n = 5 mice per group). **d**–**f** Representative immunohistochemistry images (**d**) and quantitative data (**e**,** f**) for Tregs (Foxp3^+^) and CD8^+^ T cells in the mice as described in panel a–c. Data are mean ± SEM for **b**, **c**,** e** and** f**. ** *P* < 0.01; *** *P* < 0.001; by unpaired *t* test

Furthermore, the mechanistic studies with another method of immunohistochemistry staining showed that GSK3787 treatment significantly decreased F4/80^+^ macrophages (Fig. 5a [left], b), Ly6G^+^ MDSCs (Fig. 5a [right], c), and Foxp3^+^ Tregs (Fig. 5d [left], e), but increased CD8^+^ T cells (Fig. 5d [right], f) compared to control diet treatment in *Ppard*^*TG*^ mice. Taken together, our novel findings suggested that PPARδ promoted immune suppression through upregulating Ccl20 to recruit Ccr6^+^ immunosuppressive cells (e.g., TAMs, MDSCs, Tregs) into the stomachs; GSK3787 markedly reversed the PPARδ-induced immune suppression by decreasing TAMs, MDSCs and Tregs and increasing CD8^+^ T cells in the stomachs to suppress GAC progression in *Ppard*^*TG*^ mice.

### Ccl20 is a potential circulating protein biomarker for the early detection and progression of GAC in Ppard^TG^ mice

**Fig. 6:**
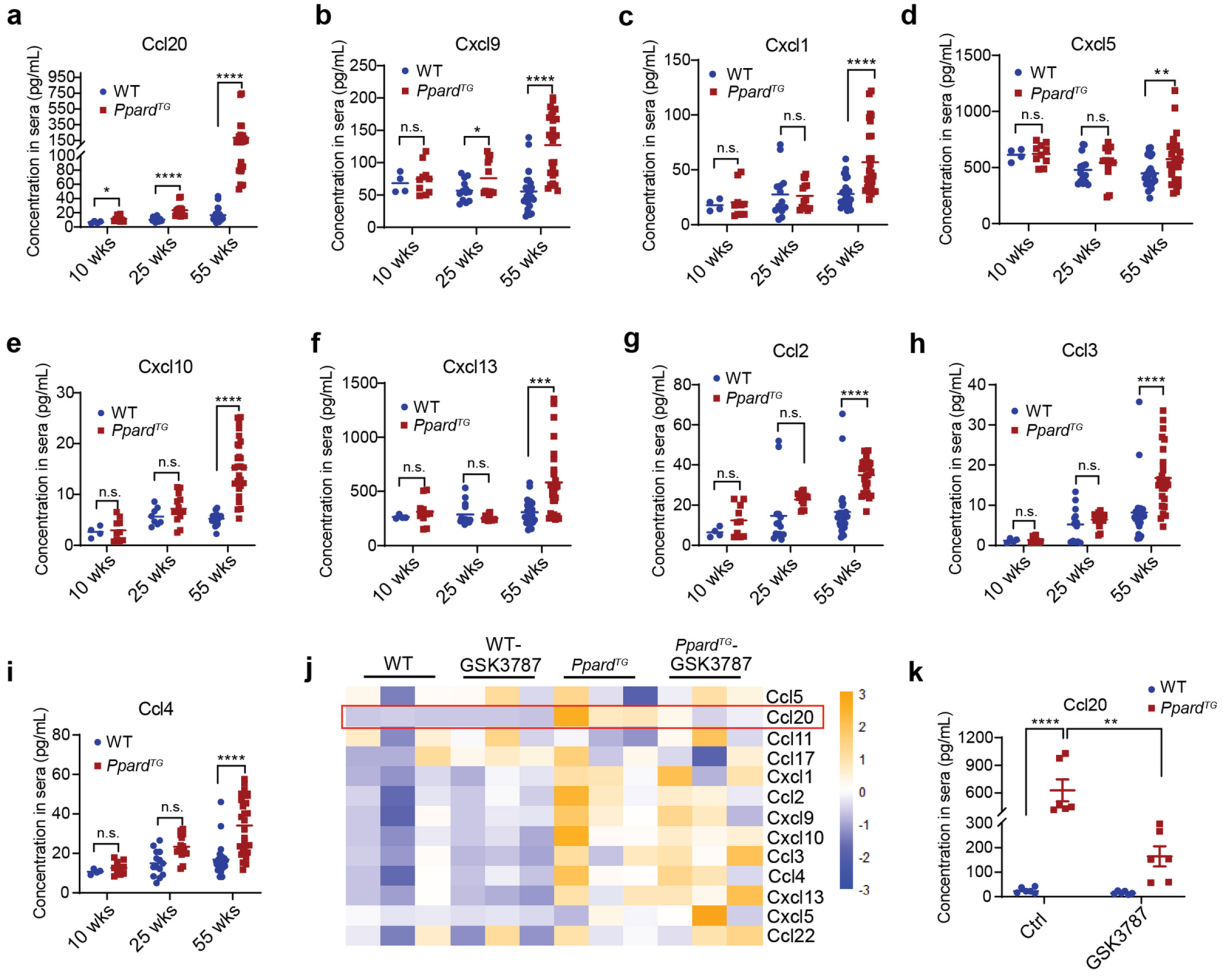
A panel of 13 chemokines were measured and compared in sera of *Ppard*^*TG*^ mice and WT littermates without or with GSK3787 treatment. **a**–**i** The sera of *Ppard*^*TG*^ and WT littermates at 10, 25, and 55 weeks were collected and measured for a panel of 13 proinflammatory chemokines using the LEGENDplex Mouse Proinflammatory Chemokine Panel kit (n = 4–10 mice for age 10 weeks, n = 14 mice for age 25 weeks, and n = 26–28 mice for age 55 weeks per group). **a**–**i** The concentrations of Ccl20 (**a**), Cxcl9 (**b**), Cxcl1 (**c**), Cxcl5 (**d**), Cxcl10 (**e**), Cxcl13 (**f**), Ccl2 (**g**), Ccl3 (**h**) and Ccl4 (**i**) in the sera of the indicated mice. **j**, **k** The sera of *Ppard*^*TG*^ and WT littermates at 6–8 weeks fed a GSK3787 or control (Ctrl) diet for 44 weeks as described in Fig. 2 were collected and measured for the same panel of 13 proinflammatory chemokines as described in panel **a**–**i** (n = 6 per group). **j** The heatmap for three representative mice per group. Heatmap was generated by R 4.2.2 ‘pheatmap’ package. **k** The concentrations of Ccl20 in the sera of the indicated mice. Data are mean ± SEM for **a**–**i**, and** k**. * *P* < 0.05; ** *P* < 0.01; ***P < 0.001; ****P < 0.0001; n.s.: not significant; by two-way ANOVA followed by Šídák’s multiple comparisons test

It is critical to identify novel biomarkers for GAC early detection and progression to improve GAC patients’ outcomes. Our *Ppard*^*TG*^ GAC mouse model recapitulating human GAC pathogenesis provides us an ideal preclinical model to identify novel biomarkers. We therefore screened for a panel of chemokines in sera of *Ppard*^*TG*^ mice and sex- and age-matched WT littermates at ages of 10 weeks (before GC development), 25 weeks (at early stages of GC, i.e., hyperplasia and low-grade dysplasia) and 55 weeks (at late stages of GC, i.e., high-grade dysplasia and adenocarcinoma) [8] using the LEGENDplex Mouse Proinflammatory Chemokine Panel, which simultaneously quantifies 13 major inflammatory chemokines (Ccl2, Ccl3, Ccl4, Ccl5, Ccl11, Ccl17, Ccl20, Ccl22, Cxcl1, Cxcl5, Cxcl9, Cxcl10, and Cxcl13). Among these chemokines, Ccl20 levels were significantly higher in sera of *Ppard*^*TG*^ mice than in those of their WT littermates at age 10 weeks, before GAC development (Fig. 6a), suggesting its potential value as an early GAC detection marker. Furthermore, Ccl20 levels continuously increased as the *Ppard*^*TG*^ mice aged from 10 to 55 weeks while the tumor progressed (Fig. 6a), suggesting the potential value of Clc20 as a GAC progression marker. The Cxcl9 levels in sera were higher in *Ppard*^*TG*^ mice at ages 25 and 55 weeks than those in their WT littermates (Fig. 6b). In addition, Cxcl1, Cxcl5, Cxcl10, Cxcl13, and Ccl2-4 levels in sera were also significantly higher in *Ppard*^*TG*^ mice than in WT littermates at age 55 weeks (Fig. 6c–i). There was no difference in serum levels of Ccl5, Ccl11, Ccl22, or Ccl17 between *Ppard*^*TG*^ mice and WT littermates at all three examined ages (Fig. S3a–d).

Interestingly, GSK3787 treatment significantly decreased Ccl20 levels in sera of *Ppard*^*TG*^ mice (Fig. 6j, k) but had no effects on the other 12 measured chemokines (Fig. S4a–l), reinforcing a critical role of PPARδ in upregulation of Ccl20 during GC tumorigenesis. Our data indicated that Ccl20 is a potential circulating biomarker for the early detection, progression of GAC, and GAC treatment surveillance in the preclinical mouse model.

**Fig. 7:**
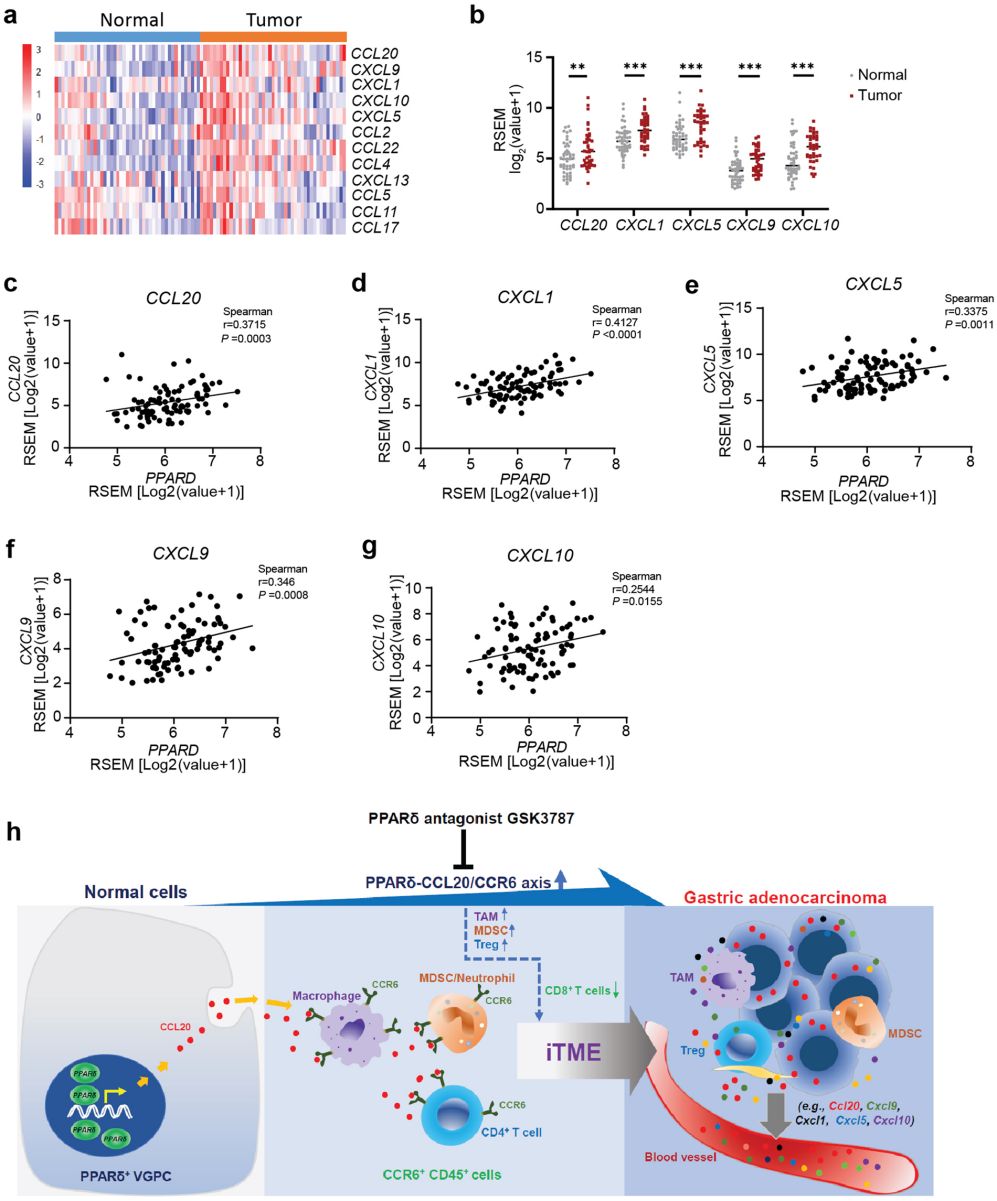
mRNA expression of the 13 chemokines in human GC and adjacent gastric normal tissues was qualified and compared, and correlations of PPARδ with the five chemokines were analyzed. **a**–**g** Human GC and adjacent gastric normal tissue mRNA expression of the 13 chemokines examined in mouse sera shown in Fig. 6 were retrieved from the dataset GSE63089/GPL5175 using the GEO2R tool and further analyzed (n = 45 paired gastric normal and tumor tissues). No data could be retrieved for *CCL3* from this database. **a**, **b** Heatmap for the 12 chemokines (**a**) and quantitative data (**b**) for the five chemokines whose mRNA expression levels were significantly higher in GC tissues than those in their adjacent gastric normal tissues. Data for **b** are mean ± SEM. **P < 0.01; ***P < 0.001; by unpaired *t* test. **c**–**g** Spearman correlation analyses of PPARδ with the 5 chemokines that were upregulated in human GCs. PPARδ was positively correlated with *CCL20* (**c**), *CXCL1* (**d**), *CXCL5* (**e**), *CXCL9* (**f**) and *CXCL10* (**g**) in gastric normal and tumor tissues (n = 90). *P* values was calculated by Spearman correlation test. RSEM: RNA-Seq by Expectation–Maximization. **h** Schematic diagram showing PPARδ overexpression in VGPC cells dysregulates CCR20/CCR6 axis to chemoattract CCR6^+^ immune cells (e.g., macrophages, MDSCs), leading to an inflammatory and immunosuppressive TME and subsequent progression of GAC carcinogenesis, while PPARδ-specific antagonist GSK3787 suppresses these tumorigenic effects

To further explore the clinical significance of these tested chemokines, we retrieved and analyzed the dataset GSE63089/GPL5175 using the NCBI GEO2R tool [26] and found that GC tissues had significantly higher mRNA expression of *CCL20, CXCL1, CXCL5, CXCL9* and *CXCL10* than those in their adjacent normal gastric tissues, but there was no significant difference in *CCL2-5, CCL11, CCL22, CXCL13*, and *CCL17* between gastric normal and tumor tissues (Fig. 7a, b). Further analyses showed that the expression levels of these identified upregulated chemokines (*CCL20, CXCL1, CXCL5, CXCL9* and *CXCL10*) in human gastric tissues of GC patients were all positively correlated with *PPARD* mRNA expression levels (Fig. 7c–g), which is consistent with our mouse findings. Taken together, both our mouse findings and human data provide a strong rationale for further studying these chemokines, particularly CCL20, in clinical applications.

## Discussion

The molecular pathogenesis of GAC, a lethal and common GC, remains poorly understood. In this study, we found that (1) PPARδ dysregulation of Ccl20/Ccr6 axis increased TAMs, MDSCs, and Tregs, but decreased CD8^+^ T cells in gastric tissues to orchestrate iTME and thus to promote GAC (intestinal-type) carcinogenesis; oral administration of GSK3787 treatment significantly suppressed the PPARδ–induced gastric immunosuppression and GAC progression; and (2) Ccl20 significantly increased as early as age 10 weeks before gastric tumor development, and its levels continuously increased with GAC (intestinal-type) progression as the mice aged from 10 to 55 weeks; oral GSK3787 treatment decreased the PPARδ-upregulated Ccl20 levels in sera of *Ppard*^*TG*^ mice.

PPARδ promotes gastric tumor stemness, inflammation, and GC tumorigenesis [8]. The PPARδ agonist GW501516 promotes DMBA-induced squamous GC in mice, a rare form of human GC [27]. Recently, PPARδ was also found to interact with Yap1 and Sox9 to promote GC malignancy [28]. An important etiological risk factor for human GAC development is an infection with *Helicobacter pylori* (*H. pylori*), a class I carcinogen [29, 30], particularly in East Asian countries [31, 32], and chronic *H. pylori* infection affects nearly half the world’s population [33]. *H. pylori or H. felis* infection induces gastric chronic inflammation while enhancing gastric stemness [34, 35]; moreover, these infections upregulate PPARδ to promote gastric epithelial proliferation in mice and humans [8, 36], indicating that PPARδ plays an important role in *H. pylori* infection-related GAC*.*

However, the etiology of the subset of GAC from populations that do not have *H. pylori* infection remains largely unclear. Our previous study showed this PPARδ-induced GAC development in *Ppard*^*TG*^ mice was unrelated to differences of stomach microbiota, indicating that this mouse model is ideal for studying pathogenesis of this subset of GAC [8]. Moreover, PPARδ expression is upregulated in human GAC and negatively associated with survival of patients with GC, particularly intestinal-type GAC, suggesting that altered PPARδ upregulation is a risk factor for GAC progression.

Although tremendous efforts have been made over many years, GC remains a life-threatening disease, with an overall 5-year survival rate less than 25% for all stages of GC and median survival less than 1 year for advanced GC [1, 2]. The poor prognosis of GC is largely due to limited therapeutic options and a lack of effective therapeutic targets. In this study, we found that transgenic overexpression of PPARδ in VGPCs resulted in GAC development and manifested as an iTME consisting of enriched TAMs, MDSCs, and Tregs, and exhausted CD8^+^ cytotoxic T cells with very low ratios of CD8^+^ cytotoxic T cells to CD4^+^ T cells by dysregulating Ccl20/Ccr6 axis. In contrast, oral administration of GSK3787 significantly inhibited GAC tumorigenesis, decreased gastric Ccl20 expression and Ccr6^+^ immune cells including TAMs, MDSCs and Tregs, and increased CD8^+^ T cell in gastric tissues. Our data suggest that PPARδ dysregulation of Ccl20/Ccr6 axis remodels iTME and thus promotes GAC carcinogenesis, thus, targeting PPARδ might be a promising therapeutic strategy for GAC patients whose tumors exhibit elevated PPARδ expression. The expansion of TAMs and MDSCs induces an iTME that promotes carcinogenesis [15–18], and low ratios of CD8^+^/CD4^+^ have been linked to poor prognosis in various human cancers including GC [37–39]. Moreover, PPARδ was found to dramatically accelerate pancreatic ductal adenocarcinoma development by activating the PPARδ-CCL2/CCR2 axis or the GOT2-PPARδ axis to drive immunosuppression through suppressing T cell-mediated anti-tumor immunity [40, 41]. Taking together our and others’ findings, it is conceivable that combination therapy with a PPARδ inhibitor/antagonist may improve the efficacy of currently available immune therapies for GAC.

Currently, many circulating tumor markers such as carcinoembryonic antigen, CA19-9, CA125, CA50, and alpha-fetoprotein have been tested in the clinic for GC detection; however, none of them has optimal sensitivity and specificity [42]. It is crucial to develop better methods for early detection of GC, given that most patients are asymptomatic until the disease progresses to advanced stages with poor prognosis. One of the significant findings of this study is the close correlation of serum Ccl20 level with GAC development and progression in preclinical mouse model. We found that among 13 examined circulating proinflammatory chemokines, Ccl20 was the only one that significantly increased in sera of the mice as early as 10 weeks before gastric premalignant lesion development, and further increased as the mice aged along with GAC development and progression. Furthermore, Ccl20 was the only marker that was significantly decreased by GSK3787 treatment. Interestingly, it has been reported that CCL20 is one of a few potential circulating markers in serum for early detection, as well as for progression of human GC in three large case-cohort studies through a high-throughput protein detection assays [43–45]. In another human study, we found that *CCL20* mRNA expression was upregulated in GC tissues, and its expression was positively correlated with *PPARD* mRNA expression [26]. Based on these findings, CCL20 may be a promising circulating biomarker for the early detection and progression of GAC and the therapeutic surveillance of PPARδ inhibitor/antagonist. However, additional factors such as GC type (e.g., intestinal-type GAC vs. diffuse GAC), tumor locations (e.g., corpus vs. antrum), and PPARδ expression levels of tumor cells (high vs. low) might need to be considered and adjusted when the significance of circulating CCL20 is evaluated as a biomarker in clinical studies.

In summary, we found that altered PPARδ-CCL20/CCR6 signaling plays a critical role in shaping an iTME and promoting GAC (intestinal-type) carcinogenesis. Our data from preclinical studies demonstrated PPARδ as a potential novel therapeutic target for GAC (intestinal-type) and CCL20 as a potential biomarker for early detection, progression, and therapeutic surveillance of GAC (intestinal-type). Further investigations are awaited to validate these findings in human studies and translate our findings to clinical applications.

### Supplementary Information

Below is the link to the electronic supplementary material.

Supplementary file1 (PDF 3002 KB)

Supplementary file2 (PDF 89 KB)

Supplementary file3 (PDF 139 KB)
