# Supplementary material for: PPARδ dysregulation of CCL20/CCR6 axis promotes gastric adenocarcinoma carcinogenesis by remodeling gastric tumor microenvironment
Source: Gastric Cancer. 2023 Aug 12;26(6):904–17. doi: 10.1007/s10120-023-01418-w (PMC10640489; doi:10.1007/s10120-023-01418-w)
Supplement: Supplementary file 1 — Supplementary file1 (PDF 3002 KB) [file 10120_2023_1418_MOESM1_ESM.pdf]

SUPPLEMENTARY FIGURES

PPARδ dysregulation of CCL20/CCR6 axis promotes gastric adenocarcinoma carcinogenesis by remodeling gastric tumor microenvironment

Journal name: Gastric Cancer

Authors: Yi Liu, Daoyan Wei, Yasunori Deguchi, Weiguo Xu, Rui Tian, Fuyao Liu, Min Xu, Fei Mao, Donghui Li, Weidong Chen, Lovie Ann Valentin, Eriko Deguchi, James C. Yao, Imad Shureiqi, and Xiangsheng Zuo

Corresponding author: Xiangsheng Zuo, Department of Gastrointestinal Medical Oncology, The University of Texas MD Anderson Cancer Center, Houston, TX 77030; xzuo@mdanderson.org.

Supplementary figure 1

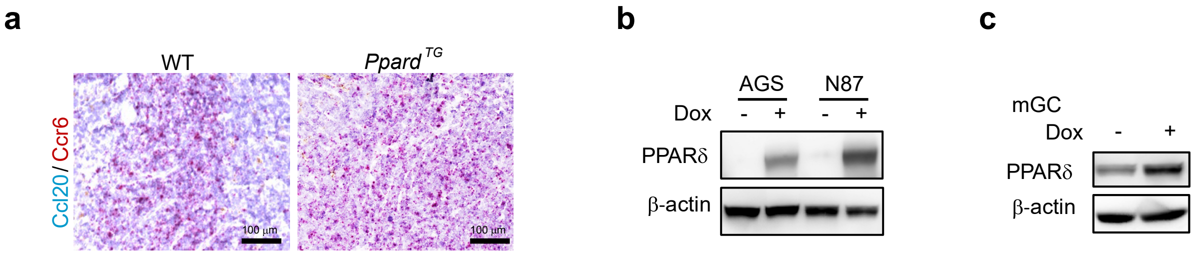

Fig. S1. *Ccl20* and *Ccr6*$^{+}$ mRNA expression was measured in the spleens of *Ppard*^{TG} and WT mice. (a) Representative images of *in situ* hybridization staining of RNAscope Duplex Assay for *Ccl20* and *Ccr6* in the spleens of *Ppard*^{TG} and WT mice at age 55 weeks. **(b, c)** PPARδ protein levels were measured by Western blot in AGS and N87 human GC **(b)** and mouse GC **(c)** cells transduced with human or mouse Dox-inducible PPARδ expression lentivirus with doxycycline (2 µg/mL) or its dissolvent treatment for 48 h.

Supplementary figure 2

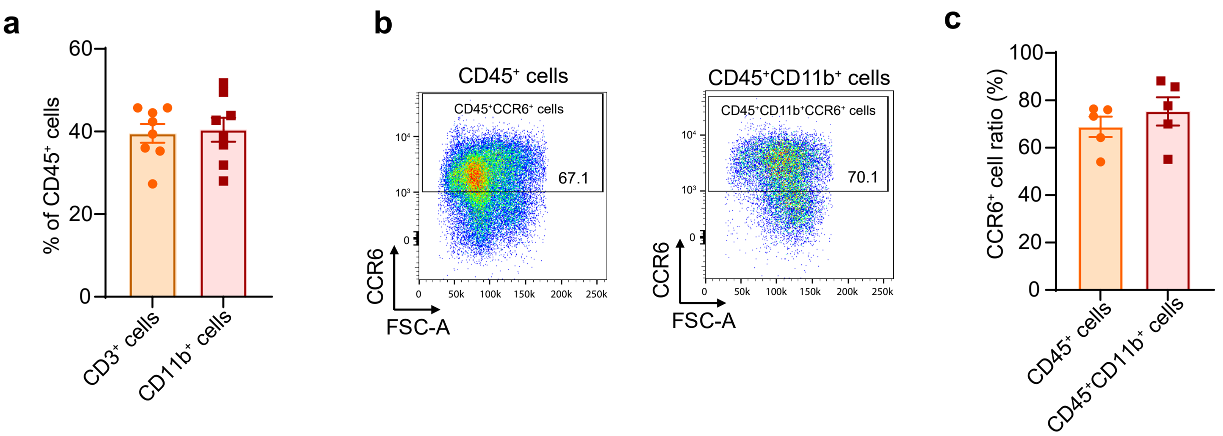

Figure S2. Subsets of stomach-infiltrating immune cells were quantified in *Ppard*^{TG} mice. (a) Percentages of stomach–infiltrating CD3^{+} T and CD11b^{+} myeloid cells out of CD45^{+} immune cells in *Ppard*^{TG} mice as described in **Figure 4** (n = 8 mice). **(b, c)** Representative flow cytometry images **(b)** and ratios **(c)** of stomach–infiltrating Ccr6^{+}CD45^{+} and Ccr6^{+}CD45^{+}CD11b^{+} myeloid cells in *Ppard*^{TG} mice (n = 5 mice).
Data are mean ± SEM for **a** and **c**.

Supplementary figure 3

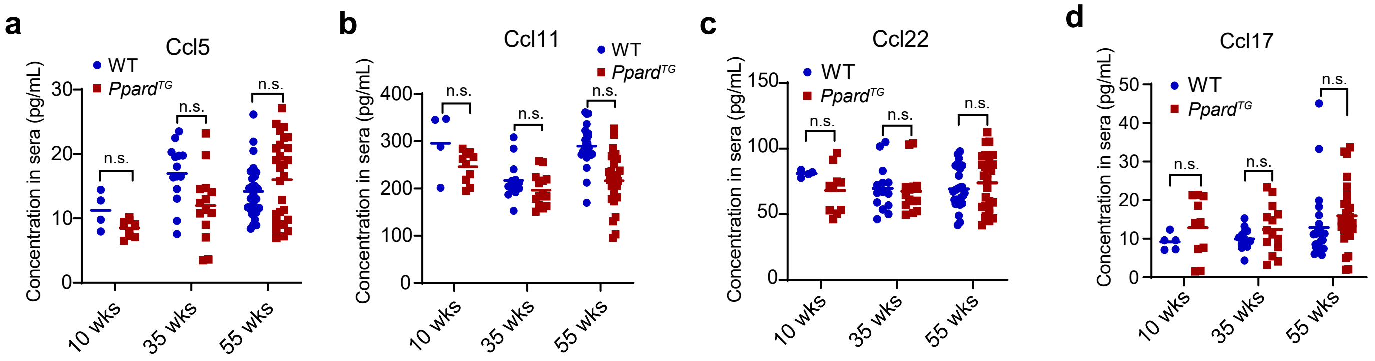

Figure S3. The chemokines were profiled and compared in sera of *Ppard*^{TG} and WT mice at three different ages. The sera of *Ppard*^{TG} mice and WT littermates at 10, 25, and 55 weeks were collected and measured for a panel of 13 proinflammation-related chemokines as described in **Figure 6a-i**. The concentrations of Ccl5 **(a)**, Ccl11 **(b)**, Ccl22 **(c)**, and Ccl17 **(d)** in the sera of the indicated mice. Data are mean ± SEM. n.s.: not significant.

Supplementary figure 4

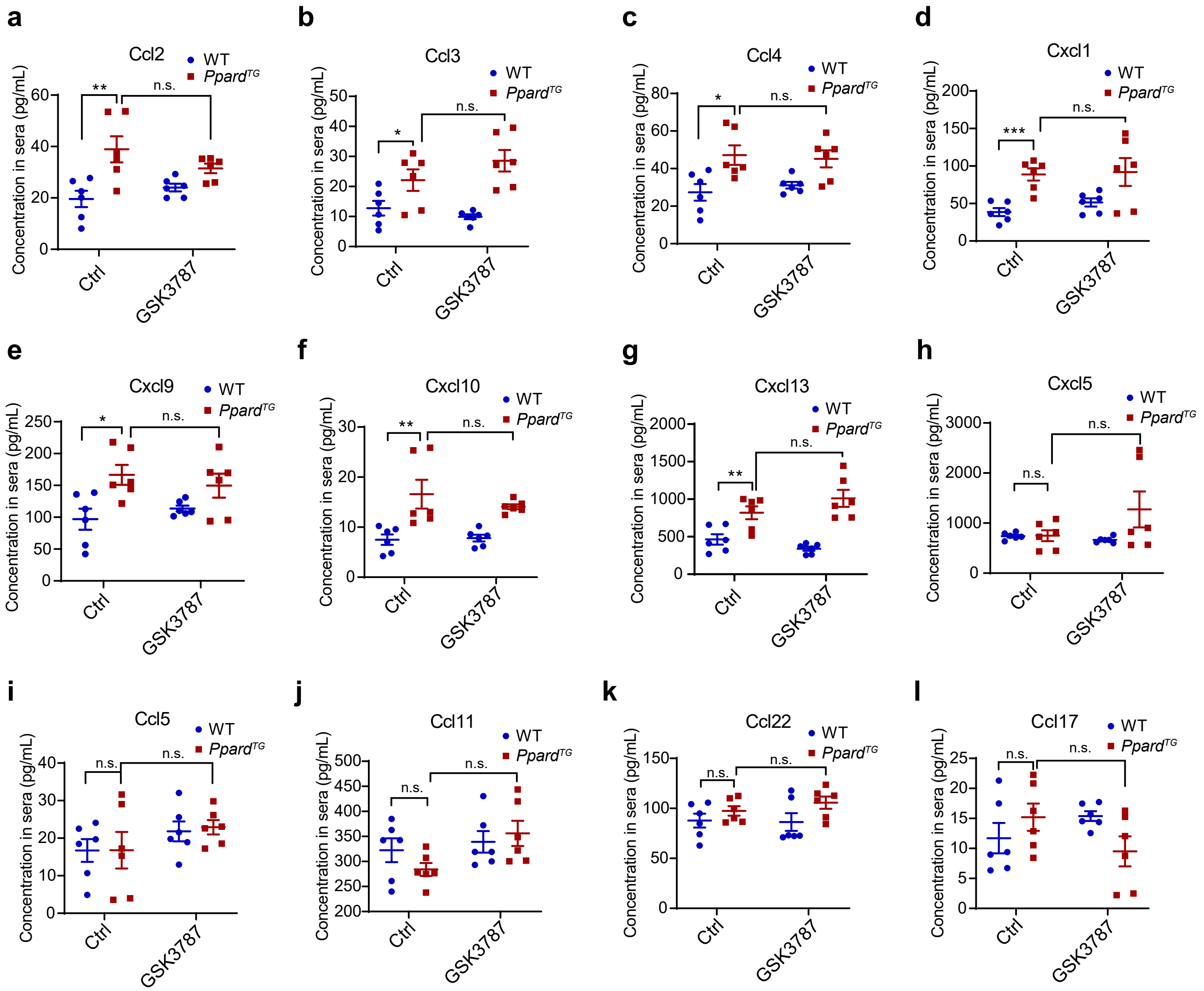

Figure S4. Effects of GSK3787 treatment on the levels of the chemokines in the sera of *Ppard*^{TG} and WT mice. The sera of *Ppard*^{TG} and WT littermates at 6-8 weeks fed a GSK3787 or Ctrl diet for 44 weeks were collected and measured for a panel of 13 inflammation-related chemokines as described in **Figure 6j, k** (n=6 per group). **(a-l)** The concentrations of Ccl2 **(a)**, Ccl3 **(b)**, Ccl4 **(c)**, Cxcl1 **(d)**, Cxcl9 **(e)**, Cxcl10 **(f)**, Cxcl13 **(g)**, Cxcl5 **(h)**, Ccl5 **(i)**, Ccl11 **(j)**, Ccl22 **(k)**, and Ccl17 **(l)** in the sera of the indicated mice.

Data are mean ± SEM. * $P < 0.05$; ** $P < 0.01$; ***$P < 0.001$; n.s.: not significant.
