# Supplementary material for: PPARδ dysregulation of CCL20/CCR6 axis promotes gastric adenocarcinoma carcinogenesis by remodeling gastric tumor microenvironment
Source: Gastric Cancer. 2023 Aug 12;26(6):904–17. doi: 10.1007/s10120-023-01418-w (PMC10640489; doi:10.1007/s10120-023-01418-w)
Supplement: Supplementary file 2 — Supplementary file2 (PDF 89 KB) [file 10120_2023_1418_MOESM2_ESM.pdf]

Table S1. Serum liver and kidney function test results for the indicated mice

Group	WT–Ctrl	WT–GSK3787	***Ppard***^{TG}**–Ctrl**	***Ppard***^{TG}**–GSK3787**
Total Protein (g/dL)	5.67 ± 0.21	5.55 ± 0.12	3.78 ± 0.23***	3.82 ± 0.61**
Albumin (g/dL)	3.60 ± 0.07	3.48 ± 0.08	2.47 ± 0.10****	2.59 ± 0.55*
Globulin (g/dL)	2.07 ± 0.18	2.07 ± 0.11	1.30 ± 0.14 **	1.23 ± 0.27*
ALP (U/L)	67.00 ± 8.19	72.67 ± 3.51	106.33 ± 23.12*	136.33 ± 23.86 **
ALT (U/L)	60.33 ± 4.73	47.67 ± 11.72	114.00 ± 27.62 *	180.33 ± 69.34 *
AST (U/L)	101.67 ± 32.72	130.33 ± 60.74	102.00 ± 33.29	211.33 ± 81.40
BUN (mg/dL)	25.53 ± 4.44	23.87 ± 0.92	25.33 ± 4.86	22.40 ± 3.40
Creatinine (mg/dL)	< 0.20	< 0.20	< 0.20	<0.20

Data are mean ± SD.* *P* < 0.05, ** *P* < 0.01, ****P* < 0.001, **** *P* < 0.0001, compared to WT–Ctrl mouse group.
