# Supplementary material for: PPARδ dysregulation of CCL20/CCR6 axis promotes gastric adenocarcinoma carcinogenesis by remodeling gastric tumor microenvironment
Source: Gastric Cancer. 2023 Aug 12;26(6):904–17. doi: 10.1007/s10120-023-01418-w (PMC10640489; doi:10.1007/s10120-023-01418-w)
Supplement: Supplementary file 3 — Supplementary file3 (PDF 139 KB) [file 10120_2023_1418_MOESM3_ESM.pdf]

PPARδ dysregulation of CCL20/CCR6 axis promotes gastric adenocarcinoma carcinogenesis by remodeling gastric tumor microenvironment

Journal name: Gastric Cancer

Authors: Yi Liu, Daoyan Wei, Yasunori Deguchi, Weiguo Xu, Rui Tian, Fuyao Liu, Min Xu, Fei Mao, Donghui Li, Weidong Chen, Lovie Ann Valentin, Eriko Deguchi, James C. Yao, Imad Shureiqi, and Xiangsheng Zuo

Corresponding author: Xiangsheng Zuo, Department of Gastrointestinal Medical Oncology, The University of Texas MD Anderson Cancer Center, Houston, TX 77030; xzuo@mdanderson.org.

Supplementary materials and methods

Antibodies

The antibodies used in this study are summarized.

Name	Source	Catalog number	Application/Dilution
Mm anti–β-Actin mAb	Santa Cruz Biotechnology	sc-47778	WB (hs/mm)/1:1000
Rb anti-PPARδ pAb	Cell Signaling Technology	74076S	WB (hs/mm)/1:1000
Rb anti-CD8α mAb	Cell Signaling Technology	98941S	IHC (mm)/1:300
Rb anti-F4/80 mAb	Cell Signaling Technology	70076S	IHC (mm)/1:300
Rb anti-FoxP3 mAb	Cell Signaling Technology	12653S	IHC (mm) /1:400
Rb anti-Ly6G mAb	Cell Signaling Technology	87048S	IHC (mm)/1:150
Rat PerCP/Cy5.5 anti-CD45 mAb	BioLegend	103131	F (mm)/1:150
Rat Brilliant Violet 785 anti-CD3ε mAb	BioLegend	100355	F (mm)/1:60
Rat PE/Cy7 anti-CD11b mAb	BioLegend	101215	F (mm)/1:250
Rat Brilliant Violet 421 anti-mouse F4/80 mAb	BioLegend	123131	F (mm)/1:400
Rat Brilliant Violet 510 anti-Ly6C mAb	BioLegend	128033	F (mm)/1:40
Rat APC/Fire 750 anti-Ly6G mAb	BioLegend	127651	F (mm)/1:80
Rat BUV737 anti-CD8α mAb	BD Biosciences	564297	F (mm)/1:200
Rat FITC anti-CD4 mAb	BioLegend	100405	F (mm)/1:200
Rat PE/Dazzle 594 anti-CCR6 mAb	BioLegend	129821	F (mm)/1:80
Rat Fluor 647 anti-Foxp3 mAb	BioLegend	126407	F (mm)/1:100
Rat Brilliant Violet 650 anti-CD25 mAb	BioLegend	102038	F (mm)/1:40
Gt anti-rb IgG HRP-linked Ab	Cell Signaling Technology	7074	WB (hs/mm)/1:2000
Gt anti-rb IgG (H+L) Ab, Biotinylated	Vector laboratories	BA-1000-1.5	IHC (mm)/1:300

Abbreviations: mm=mouse; hs=human; gt=goat; rb=rabbit; mAb=monoclonal antibody; pAb=polyclonal antibody; IHC=immunohistochemistry; F=flow cytometr

Evaluation of potential GSK3787 side effects

To evaluate the potential side effects of the GSK3787 diet treatment on the mice, total protein, albumin and globulin, ALP, ALT and AST for liver function and BUN and Creatinine for kidney function in the sera of the *Ppard*^{TG} mice and their control WT littermates fed control diet or GSK3787 diet for 44 weeks, were measured in MD Anderson Veterinary Pathology, Hematology and Chemistry Core Facility by using Cobas Integra 400 Plus Chemistry Analyzer from Roche Diagnostics.

Immunohistochemistry staining

Immunohistochemistry staining was performed similarly as described before (1). Briefly, tissue sections (5µm thick) were deparaffinized and rehydrated. Antigen retrieval was then performed by immersion of slides into Antigen Unmasking Solution (#H-3300, Vector Laboratories) and heated in a steam chamber for 35 min. Slides were treated with 3% H_2O_2 solution to reduce endogenous peroxidase, incubated with blocking buffer (5% goat serum in Tris-buffered saline with 0.1% Tween 20) for 30 min, and then incubated with primary antibodies overnight. The following primary antibodies were used: Ly6G (#87048, 1:150), CD8a (#98941, 1:300), F4/80 (#70076, 1:300), and Foxp3 (#12653, 1:400) from Cell Signaling Technology. On the second day, the tissue sections were incubated with biotinylated secondary antibodies (#BA-1000; Vector Laboratories) for 1 h, followed by incubation with avidin-coupled peroxidase (Vector Laboratories) for 30 min. The slides were developed using 3,3'-diaminobenzidine (DAB) (Agilent Dako) and then counterstained with Mayer's hematoxylin (Agilent Dako). The positive staining cells were counted under microscope with 40X magnification for 5 randomly selected fields, then averaged and presented as quantitative data for each mouse.

Generation of human and mouse GC cell lines with Doxycycline-inducible Tet-on PPARD expression

The human gastric cancer cell lines AGS and N87 were purchased from ATCC; Mouse GC cell line was generated from GC tissues of 55 weeks-old *Ppard*^{Tg} mice and further characterized as described before (2). All three cell lines were cultured in high glucose DMEM medium supplemented with 10% fetal bovine serum (VWR International), 2 mM L-glutamine, and 1% penicillin/streptomycin (Life Technologies).

Human or mouse PPARD ORF was subcloned into pLVX-TetOne-Puro Doxycycline-inducible expression lentiviral vector (Cat#631849, Takara Bio USA, Inc) and then the human and mouse PPARD lentiviral inducible expression vectors were packaged into lentiviral particles at MD Anderson's shRNA and ORFeome Core Facility. Human GC cell lines AGS and N87 or mouse GC cells (2) were transduced with human or mouse PPARD lentiviral particles (10 MOIs for all used cell lines) with hexadimethrine bromide (8 µg/mL), respectively. After 12 h, the culture medium was replaced with fresh medium containing puromycin (2 µg/mL). The medium was changed once every 72 h. The cells that survived from puromycin selection were expanded for further analyses.

RNA extraction and quantitative reverse transcription PCR (RT-qPCR)

For extraction of total RNA, the scraped gastric epithelial cells were quickly homogenized in TRIzol by 1-ml syringe with #25 Gauge needle. Total RNA was isolated according to the TRIzol manufacturer's instruction. mRNA relative expression levels were measured by RT-qPCR as described before, and the mouse beta-actin (*Actb*) and human HPRT gene was used as mouse and human endogenous control, respectively (3, 4).

TaqMan expression probes' information for qPCR is summarized.

**Name**	**Source**	**Assay ID or Catalog number**
Mouse Ccl20 (FAM/MGB)	Thermo Fisher Scientific	Mm01268754_m1
Mouse Ifng (FAM/MGB)	Thermo Fisher Scientific	Mm01168134_m1
Mouse Actb (FAM/ZEN/IBFQ)	Integrated DNA Technologies	Mm.PT.39a.22214843.g
Human CCL20 (FAM/MGB)	Thermo Fisher Scientific	Hs00355476_m1
Human HPRT1 endogenous control (VIC/MGB)	Thermo Fisher Scientific	4326321E

Protein lysate preparation and Western blot analysis

Protein lysate preparation and Western blot was performed as described before (5). Briefly, cells were homogenized in lysis buffer comprising 1% SDS, 0.5% Nonidet P-40, 20 mM 3-(N-morpholino) propanesulfonic acid (pH 7.0), 2 mM ethylene glycol tetraacetic acid, 5 mM EDTA, 30 mM sodium fluoride, 40 mM β-glycerophosphate, 2 mM sodium orthovanadate, 1mM phenylmethylsulfonyl fluoride (all from Sigma-Aldrich) and 1× cOmplete protease inhibitor cocktail (Roche). Forty-microgram protein lysates per sample were separated in 12% sodium dodecyl sulfate–polyacrylamide gel by electrophoresis, and then transferred to a 0.2 μm nitrocellulose membrane. The membranes were blocked with 5% milk for 2 h at room temperature and hybridized with the primary antibody at 4°C overnight. The primary antibodies used were as follows: PPARδ (#74076, Cell Signaling Technology), β-actin (#sc-47778, Santa Cruz Biotechnology). Then, the blots were hybridized with the secondary antibody for 1 h at room temperature. The blots were analyzed using enhanced chemiluminescence (#170-5061, Bio-rad).

Immune cell profiling by flow cytometry

Immune cell profiling by flow cytometry was performed as described before (1, 2). Briefly, around 200 mg of mouse stomach tissues were cut into 1-mm^3 pieces. Mouse stomach tissues were then digested in 10 ml of digestion buffer (1 mg/mL collagenase IV [#C-5138, Sigma-Aldrich], 0.1 mg/mL hyaluronidase V [#H-6254, Sigma-Aldrich] and 30 U/mL DNase I [#D-5025, Sigma-Aldrich] in Dulbecco's Modified Eagle Medium) at 37°C for 60 min with shaking at 150rpm. The digested tissues were pushed through a 70-μm cell strainer and washed by 40 ml of Dulbecco's Modified Eagle Medium without fetal bovine serum. The digested cells were re-suspended in 37% Percoll, with the same volume of 70% Percoll on the bottom of the tube. After centrifugation at 800*g* for 20 min with brake off, the immune cells were isolated and collected from the 37%/70% interface and rinsed by phosphate-buffered saline. The isolated gastric immune cells were first stained by Zombie UV (#423107, BioLegend), and then incubated with a cocktail comprising the following antibodies in cell staining buffer (#420201, BioLegend): anti-CD45 (#103131), anti-CD3ε (#100355), anti-CD11b (#101215), anti-F4/80 (#123131), anti-Ly6C (#128033), anti-Ly6G (#127651), anti-CD4 (#100405), anti-Foxp3 (#126407), anti-CD25 (#102051), and anti-CCR6 (#129821),

all from BioLegend, and anti-CD8a (#564297, BD Biosciences). After washing, the stained cells were submitted for multiple-color flow cytometry analysis. The data were collected on LSR Fortessa X-20 analyzer with FACSDiva software version 8.0 (BD) and analyzed using FlowJo version 10 (BD).

Quantification of a panel of 13 LEGENDplex mouse proinflammatory chemokines in sera

The panel profiling of proinflammatory chemokines (#740007, BioLegend) in mouse sera was performed according to the manufacturer's protocol as described before (2). The assay uses the principles of a sandwich enzyme-linked immunosorbent assay to quantify soluble analytes using a flow cytometer, which allows simultaneous quantification of 13 mouse chemokines (Ccl2, Ccl3, Ccl4, Ccl5, Ccl11, Ccl20, Ccl22, Cxcl1, Cxcl5, Cxcl9, Cxcl10, Cxcl13, and Ccl17). The data were analyzed using the LEGENDplex v8.0 Data Analysis Software. The results were presented as picograms per milliliter of sera as described before (2).

References

1. Liu Y, Deguchi Y, Wei D, Liu F, Moussalli MJ, Deguchi E, et al. Rapid acceleration of KRAS-mutant pancreatic carcinogenesis via remodeling of tumor immune microenvironment by PPARδ. Nat Commun. 2022;13(1):2665. Epub 2022/05/14. doi: 10.1038/s41467-022-30392-7. PubMed PMID: 35562376; PubMed Central PMCID: PMCPMC9106716.
2. Zuo X, Deguchi Y, Xu W, Liu Y, Li HS, Wei D, et al. PPARD and Interferon gamma Promote Transformation of Gastric Progenitor Cells and Tumorigenesis in Mice. Gastroenterology. 2019. Epub 2019/03/20. doi: 10.1053/j.gastro.2019.03.018. PubMed PMID: 30885780.
3. Yan Z, Gao J, Lv X, Yang W, Wen S, Tong H, et al. Quantitative Evaluation and Selection of Reference Genes for Quantitative RT-PCR in Mouse Acute Pancreatitis. Biomed Res Int. 2016;2016:8367063. Epub 2016/04/14. doi: 10.1155/2016/8367063. PubMed PMID: 27069927; PubMed Central PMCID: PMCPMC4812220.
4. Liu Y, Deguchi Y, Tian R, Wei D, Wu L, Chen W, et al. Pleiotropic Effects of PPARD Accelerate Colorectal Tumorigenesis, Progression, and Invasion. Cancer Res. 2019;79(5):954-69. Epub 2019/01/27. doi: 10.1158/0008-5472.Can-18-1790. PubMed PMID: 30679176; PubMed Central PMCID: PMCPMC6397663.
5. Liu F, Zuo X, Liu Y, Deguchi Y, Moussalli MJ, Chen W, et al. Suppression of Membranous LRP5 Recycling, Wnt/β-Catenin Signaling, and Colon Carcinogenesis by 15-LOX-1 Peroxidation of Linoleic Acid in PI3P. Cell Rep. 2020;32(7):108049. Epub 2020/08/20. doi: 10.1016/j.celrep.2020.108049. PubMed PMID: 32814052; PubMed Central PMCID: PMCPMC8765570.
